# Identification of Key Differentially Expressed Genes During Early Sex Determination in Chicken Embryos

**DOI:** 10.3390/ijms26199575

**Published:** 2025-10-01

**Authors:** Ruijia Liu, Huanhuan Miao, Bo Zhang, Hao Zhang

**Affiliations:** 1 Frontiers Science Center for Molecular Design Breeding (MOE), China Agricultural University, Beijing 100193, China; s20233040754@cau.edu.cn (R.L.); zhanghao827@163.com (H.Z.); 2 National Engineering Laboratory for Livestock and Poultry Breeding, Beijing Key Laboratory of Animal Genetic Engineering, China Agricultural University, Beijing 100193, China; 15175537700@163.com

**Keywords:** chicken embryonic gonad, sex determination, transcriptome, gonadal differentiation

## Abstract

In the chicken industry, sex determination significantly affects production efficiency and raises ethical concerns in poultry farming. As a key economic species, maximizing the advantages of each sex is vital in modern intensive breeding. Therefore, understanding the mechanisms of sex determination and regulation is critical to advancing the poultry industry. Transcriptome analysis of 3.5-day-old White Leghorn chicken embryonic genital ridges (*n* = 30, 15 males and 15 females) was performed using sex-pooled samples (five embryos/replicate, three replicates/sex). Sequencing generated 39.6 GB of high-quality reads for inter-sex comparative analysis, revealing 283 significantly differentially expressed genes (DEGs). The DEGs were primarily enriched in pathways such as ribosome biogenesis, glycan biosynthesis and metabolism, and TGF-β signaling, which are potential candidate pathways for the differentiation of chicken embryonic gonads. Key DEGs (including *SMAD2Z*, *FREM1*, *NR2F1*, *SEMA6A*, *NFIB*, *RNF165*, *SMAD7B*, *SMAD2W*, *SPIN1W*, and *HINTW*) were validated by RT-qPCR, confirming the transcriptome sequencing results. Among the DEGs, we predict binding sites for *NR2F1* and *NFIB* within the *DMRT1* gene promoter and suggest that these factors may serve as potential upstream activators for the expression of *DMRT1*, and they may initiate high *DMRT1* expression in the subsequent stages of male embryos and regulate testicular development. In conclusion, this study investigated DEGs in the gonads of male and female chicken embryos after 3.5 days of incubation and found that *NR2F1* and *NFIB* may serve as potential upstream activators for the expression of *DMRT1*, which is involved in the early determination of chicken sex.

## 1. Introduction

Both birds and mammals rely on genetic sex determination through sex chromosomes, but the regulatory genes differ. In early stages of the embryo once sex differentiation begins, specific genes and hormones guide gonadal development into ovaries or testes. Mammals use the XX/XY sex chromosome system, with the *SRY* (sex-determining region Y) gene on the Y chromosome triggering male development. In contrast, chickens use a ZZ/ZW sex chromosome system, where ZW embryos develop as females [1], and their gonads exhibit asymmetrical development [2]. Although several sex determination-related genes have been found in chickens, they are not expressed in the same pattern as in mammals [3].

Several hypotheses have been proposed regarding sex determination in chickens. Sex determination is influenced by Z-linked gene dosage, as proposed by the Z chromosome dosage hypothesis, with males possessing higher expression levels [4,5]. *DMRT1*, a candidate sex determinant in the Z-dosage effect hypothesis, plays a role in sex determination and gonadal development across various species [6]. *DMRT1* is a key regulator of sex determination in chickens, and its transcription is regulated by a long noncoding RNA derived from the MHM locus [7,8]. Testicular development is strongly associated with *DMRT1* expression in chicken embryonic gonads. However, other sex-linked genes might act upstream and autonomously to determine sex, implying that *DMRT1* could serve as a target for these yet-to-be-defined genes within the gonad [9]. The sex of all avian species is genetically determined, potentially influenced by the dosage of genes linked to the Z chromosome, the dominant effects of genes determining ovarian development located on the W sex chromosome, or a combination of these factors. These determinants may exert their influence prior to the clear differentiation of the gonads into either testes or ovaries, as well as in tissues beyond the urogenital system [10]. Nonetheless, prevailing theories have yet to comprehensively account for the mechanisms underlying sex selection in avian species, highlighting the complexity and need for further investigation of this biological phenomenon.

Chickens represent economically significant animals and unique model organisms and thus are central to studying the mechanisms of sex determination in birds. The ability to control and select the sex of poultry can substantially reduce breeding costs in the poultry industry. Currently, research on chicken sex determination focuses mainly on the period after E4.5 when the gonads become obviously differentiated [11,12,13]. However, as primordial germ cells (PGCs) enter the gonads at E3, they already exhibit an initial sex identity [12,14,15]. Therefore, at the subsequent E3.5 stage, although male and female gonads do not visibly differ, certain genes must act as potential upstream activators for the activation of sex determination and gonadal differentiation. Although *DMRT1* has been established as a decisive factor for testis versus ovary differentiation that acts at E6, it is not differentially expressed at the earlier gonad-forming stage E3.5 [7,11]. Thus, the upstream genes that trigger Sex determination and initiate gonadal differentiation prior to E3.5 remain unknown.

Therefore, in this experiment, chickens were used as the model and transcriptome sequencing technology was employed to investigate the expression profiles of genes involved in sex determination at the E3.5 stage of embryonic development. The findings are expected to lay a solid foundation for further understanding vertebrate evolution and determining the molecular mechanisms underlying sex determination and differentiation. Moreover, they will provide a theoretical and practical basis for exploring sex control in chickens and guiding the development of animal husbandry.

## 2. Results

### 2.1. High-Throughput Sequencing and Read Mapping

To reveal the mechanisms underlying the differences in chicken embryo gonad differentiation, RNA-seq was conducted to study the gene expression profiles at the transcriptional level. RNA-seq libraries were prepared from the same side of both female and male 3.5-day-old chicken embryos and sequenced on the Illumina platform. This yielded 20.6–22.5 million clean reads for female chicken embryo gonads and 20.4–22.3 million clean reads for male chicken embryo gonads. Over 90% of the reads were mapped, and more than 18,000 genes were identified in all samples (Table 1). These results demonstrated the reliability of our data.

### 2.2. RNA-Seq and Data Analysis

**Figure 1:**
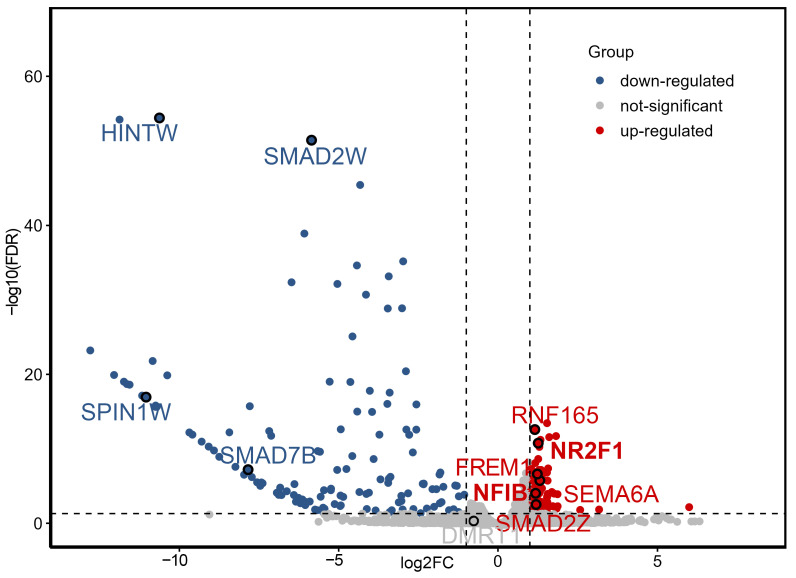
Volcano plot of differentially expressed genes between male and female embryonic gonads at E3.5. Red points indicate genes upregulated in male gonads (log2FC > 1, FDR < 0.05); blue points indicate genes downregulated in male gonads (log2FC < −1, FDR < 0.05). Dashed lines indicate the significance thresholds: |log2FC| > 1 and FDR < 0.05.

To determine which genes play a regulatory role in gonadal development in chick embryos by 3.5 days, we analyzed the DEGs between male and female gonads at this stage. A total of 283 DEGs were obtained from the gonads of female and male embryos analyzed at 3.5 days (DEGs were defined as |log2FC| > 1 and FDR [Benjamini–Hochberg adjusted *p*-value] < 0.05). Of these, 143 DEGs were upregulated and 140 were downregulated in male chicks (Figure 1). The DEGs showed that the expression had good repeatability in female and male chicken embryo groups. We focused on several key differentially expressed genes: those upregulated in male chickens (*SMAD2Z*, *FREM1*, *NR2F1*, *SEMA6A*, *NFIB*, and *RNF165*) are located on the Z chromosome, whereas four genes upregulated in females (*SMAD7B*, *SMAD2W*, *SPIN1W*, and *HINTW*) are located on the W chromosome.

### 2.3. Gene Ontology, KEGG Pathway and GSEA Analysis

**Figure 2:**
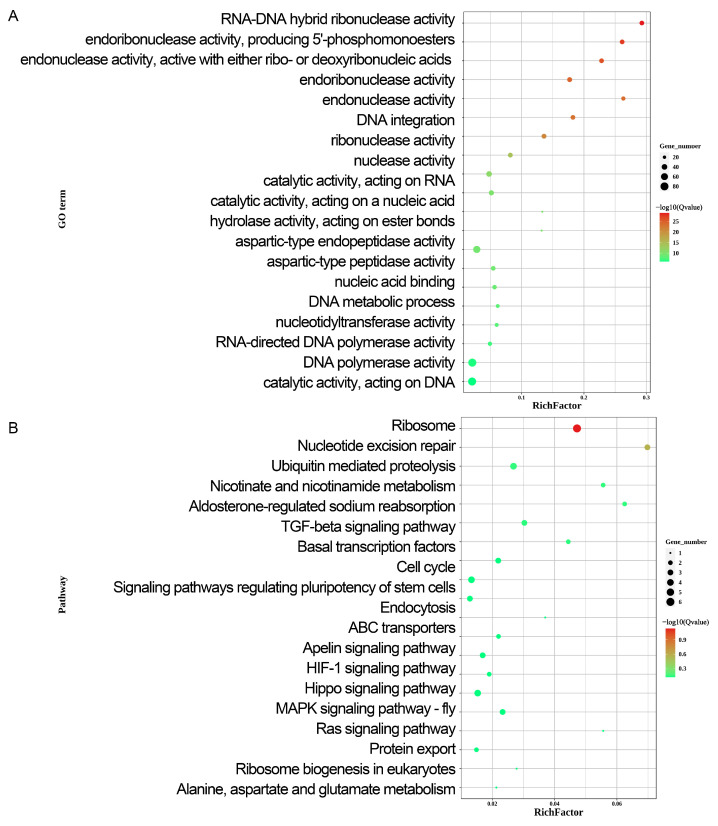
GO and KEGG enrichment analysis of DEGs between male (ZZ) and female (ZW) embryonic gonads at E3.5 (Gallus gallus). (**A**) GO analysis of DEGs. Enrichment plot of GO entries is shown. Different colors represent −log10(Q-value), and circle size indicates the number of differentially expressed genes. (**B**) KEGG analysis of DEGs. KEGG summary map of KEGG pathways. Different colors represent −log10(Q-value), and circle size indicates the number of differentially expressed genes.

To further assess the functional role of DEGs in early sex determination, GO enrichment analysis was performed (Figure 2A, Supplementary Table S2). The analysis revealed that the DEGs were primarily involved in cellular processes, metabolic processes, bioregulation, regulation of biological processes, stimulus responses, signaling, multicellular organismal processes, positive regulation of biological processes, developmental processes, negative regulation of biological processes, biological processes of inter-species interactions, reproduction processes, and immune system processes. Figure 2A illustrates the GO enrichment classification and the top 20 most significantly enriched GO terms. Additionally, DEGs that were highly expressed in females (Supplementary Table S3) were primarily enriched in binding, catalytic activity, ATP-dependent activity, molecular function regulation, transcriptional regulatory activity, molecular transduction activity, developmental processes, reproduction, reproductive processes, and responses to stimuli. In contrast, DEGs that were highly expressed in males (Supplementary Table S4) were primarily enriched in binding, catalytic activity, transcriptional regulatory activity, antioxidant activity, developmental processes, growth, reproduction, and reproductive processes.

To better understand the biological functions and interactions of the DEGs, KEGG pathway analysis was performed on the identified DEGs (Figure 2B, Supplementary Table S2). This analysis revealed that the DEGs were primarily associated with genetic information processing, cellular processes, environmental information processing, and metabolism pathways, all of which are integral components of complex biological systems. Figure 2B illustrates the KEGG enrichment analysis and top 20 most significantly enriched KEGG signaling pathways. The KEGG signaling pathway enrichment analysis revealed that DEGs in females (Supplementary Table S3) were primarily associated with the signal transduction, folding, sorting and degradation, translation, and amino acid metabolism pathways. In contrast, this analysis revealed that in DEGs in males (Supplementary Table S4) were mainly associated with the polysaccharide biosynthesis, metabolism, and energy metabolism pathways. The current study focused on sex chromosome-located DEGs (*SMAD2Z*, *FREM1*, *NR2F1*, *SEMA6A*, *NFIB*, *RNF165*, *SMAD7B*, *SMAD2W*, *SPIN1W*, *HINTW)*. GO and KEGG results indicate that by embryonic E3.5, male and female gonads already exhibit significant sex differences in protein synthesis and metabolic regulation; this finding is corroborated by GSEA (Supplementary Figure S1). KEGG pathway enrichment analysis indicated that these genes are involved in cell adhesion and migration (*FREM1*, *SEMA6A*, *NFIB*), ubiquitin-mediated protein degradation (*RNF165*), environmental signal response (*SEMA6A*, *RNF165*), and TGF-β signaling (*SMAD7B*, *SMAD2W*, *SMAD2Z*).

### 2.4. qPCR Results at DEGs

**Figure 3:**
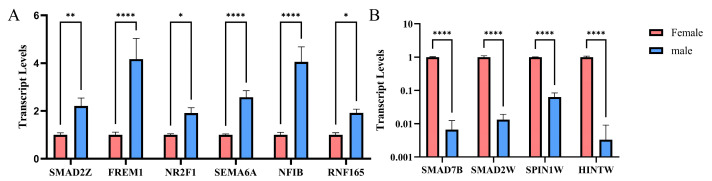
qRT-PCR validation of transcript levels of key differentially expressed genes in male and female chicken embryos at E3.5. (**A**): Genes highly expressed in males; (**B**): Genes highly expressed in females. * *p* < 0.05, ** *p* < 0.01, **** *p* < 0.0001.

To explore the transcriptomics-selected DEGs related to reproductive development, a qRT-PCR analysis on ten DEGs located on sex chromosomes related to reproductive development was performed: *SMAD2Z*, *FREM1*, *NR2F1*, *SEMA6A*, *NFIB*, *RNF165*, *SMAD7B*, *SMAD2W*, *SPIN1W*, and *HINTW* (Figure 3). It was speculated that these genes would be involved in the sex determination process in chickens. Among them, *SMAD2Z*, *FREM1*, *NR2F1*, *SEMA6A*, *NFIB*, and *RNF165* showed significantly higher expression levels in male gonads than in female gonads, whereas the expression levels of *SMAD7B*, *SMAD2W*, *SPIN1W*, and *HINTW* were significantly higher in female gonads than in male gonads.

### 2.5. Prediction of Binding Between Transcription Factors NFIB and NR2F1 with DMRT1

**Figure 4:**
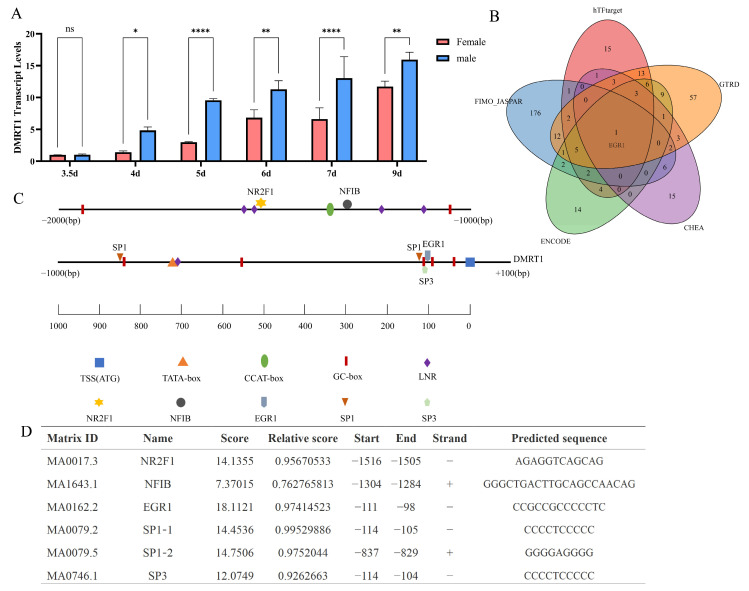
Gene expression and transcription factor matching scores and binding sites. (**A**) Relative expression of the *DMRT1* in female and male chicken embryos during the sex determination process. (**B**) Venn diagram showing predicted human *DMRT1* transcription factors from hTFtarget, JASPAR, ENCODE, ChEA and GTRD databases. Numbers indicate the number of shared transcription factors. (**C**) Schematic representation of the chicken *DMRT1* promoter region. The transcription start site (TSS) is set as position 0. The diagram shows the –2000 to +100 bp region relative to the TSS. LNR: initiator (**D**) Predicted transcription factor binding sites and their scores for the chicken DMRT1 gene. * *p* < 0.05; ** *p* < 0.01; **** *p* < 0.0001; ns: not significant (*p* ≥ 0.05).

*DMRT1*, a crucial candidate gene for avian sex determinatio [6], was not differentially expressed in the transcriptomes of 3.5-day-old chicken embryo gonads. This result was also confirmed by qPCR validation (Figure 4A). *DMRT1* was highly expressed in males after 4 days of incubation (Figure 4A). This suggests that during the initial 3.5 days of development of chicken embryos, other genes may be differentially expressed to contribute to the regulation of *DMRT1* expression, thereby modulating the development of embryo gonads and ultimately influencing chicken sex determination. *NR2F1* and *NFIB*, which are highly expressed in 3.5-day-old males, are transcription factors, and binding sites to the *DMRT1* promoter region are predicted to yield one transcription factor binding site each. Due to the lack of a chicken transcription factor database, we queried the hTFtarget, JASPAR, ENCODE, ChEA, and GTRD databases for transcription factors of human *DMRT1*, and the intersection of these predictions yielded *EGR1* (Figure 4B). Notably, previous studies have shown that *SP1*, *SP3* and *EGR1* act as transcriptional regulators of the rat *Dmrt1* [16]. In Figure 4C, we present a schematic of the chicken *DMRT1* promoter region, indicating the transcription start site and locations of core regulatory elements, with predicted binding sites annotated for *NR2F1* and *NFIB* (from our transcriptome results) as well as for the identified key transcription factors *SP1*, *SP3* and *EGR1*. Figure 4D displays the predicted binding site locations within the chicken *DMRT1* promoter and their corresponding scores. These results indicate that *NR2F1* is positioned near the LNR (Initiator) element of the *DMRT1* promoter, *NFIB* is close to the CCAT-box, and *SP1*, *SP3* and *EGR1* are adjacent to the GC-box. We also predicted all transcription factors and their potential binding sites within the core promoter region of the chicken DMRT1 gene with relative scores above 0.80 (Supplementary Table S5). These genes may act as transcriptional regulators of *DMRT1* during early embryogenesis and could contribute to its upregulation in subsequent developmental stages, thereby promoting the differentiation of the genital ridge toward testicular fate.

## 3. Discussion

In this study, transcriptome sequencing was conducted on the sexual glands of female and male chicken embryos at the E3.5 stage with the aim of identifying potential upstream activators for chicken sex determination during the early undifferentiated sex period. Expression levels were normalized to fragments per kilobase of transcript per million mapped reads (FPKM). Differentially expressed genes (DEGs) between male and female embryonic gonads at E3.5 were identified using thresholds of |log2 fold change| > 1 and FDR (Benjamini–Hochberg adjusted *p*-value) < 0.05. Transcriptome screening of E3.5 gonads revealed ten key DEGs located on the sex chromosomes with FPKM > 20: *SMAD2Z*, *FREM1*, *NR2F1*, *SEMA6A*, *NFIB*, *RNF165*, *SMAD7B*, *SMAD2W*, *SPIN1W*, and *HINTW*. These genes showed significant differential expression between sexes in the early stages prior to gonadal differentiation. Thus, these genes are likely key candidate genes involved in gonadal determination and differentiation in chickens. Upregulated genes in male chickens (*SMAD2Z*, *FREM1*, *NR2F1*, *SEMA6A*, *NFIB*, and *RNF165*) were located on the Z chromosome, whereas four genes upregulated in females (*SMAD7B*, *SMAD2W*, *SPIN1W*, and *HINTW*) were located on the W chromosome. Analysis of the gonads of male and female chicken embryos incubated for 3.5 days via transcriptome sequencing revealed that the DEGs were mainly enriched in the TGF-β signaling, energy metabolism, and ribosome synthesis pathways. *SMAD7B*, *SMAD2W*, and *SMAD2Z* were all involved in the transforming growth factor β (TGF-β) signaling pathway. TGF-β regulates cellular behavior in both embryonic and adult tissues by binding to plasma membrane serine/threonine kinase receptors, which activates Smad molecules and other signaling proteins to collectively control gene expression [17]. This pathway participates in various biological processes, including tissue and organ growth and development, immune responses, and reproductive development [18,19,20]. In adult mammals, the TGF-β signaling pathway regulates the growth and differentiation of gonadal somatic cells and germ cell [21]. Experiments in mice have further confirmed the effect of the TGF-β signaling pathway on animal reproduction [22]. Therefore, during the early stages of chicken embryo incubation, the TGF-β signaling pathway may regulate the expression of genes related to sex determination and differentiation, leading the chicken embryo towards female or male differentiation. Given their significant differential expression in the gonads of chickens of different sexes, *SMAD7B*, *SMAD2W*, and *SMAD2Z* likely play important regulatory roles in gonadal differentiation. *RNF165* may contribute to this process by enhancing the transcriptional activity of SMAD family effectors and promoting the ubiquitination and degradation of SMAD inhibitors [23]. *FREM1* encodes extracellular matrix proteins and is involved in gonadal sexual differentiation in Leopard Geckos [24]. *HINTW* is located on the chicken W chromosome [25] and expressed in both the gonadal and urogenital tracts, and it is highly expressed in female chicken embryos [26]. Further studies have shown that *HINTW* plays a significant role in chicken feminization and ovarian development [18]. However, these genes only influence the chicken sex determination process and do not represent critical sex switching genes; therefore, further research is required to reveal the most critical upstream sex determination switch genes.

Nuclear receptor subfamily 2 group F member 1 (*NR2F1*, or *COUP-TFI*) is an orphan nuclear receptor because the ligands for *NR2F1* have not been identified. *NR2F1* functions as a homodimer and is one of the major transcriptional regulators that direct cortical arealization, cell differentiation, and maturation [27,28,29]. Although NR2F1’s role in sex determination has not been investigated, *NR2F1* and its homolog *NR2F2* exhibit high structural similarity, DNA-binding properties, functional roles, and regulatory roles. Studies have demonstrated their functional interchangeability when coexpressed in the same cell [30]. A recent study combining single-nucleus multiomics and *NR2F2* ChIP-seq in mice demonstrated that *NR2F2* plays a critical role in establishing mesenchymal cell identity, fetal Leydig cell differentiation, and overall testicular morphogenesis and is an important regulator of sexual development [31]. *NR2F2* is also expressed in theca cells and uterine tissue and plays a significant role in female reproduction. It can be hypothesized that *NR2F1* might have a similar function in gonadal development in chickens. Nuclear factor I B (*NFIB*) transcription factors of the nuclear factor I (NFI) family are critical regulators of cellular differentiation, proliferation, and homeostatic maintenance. By interacting with different transcription factors, NFIs contribute to the co-regulation of broad-spectrum cellular processes and signaling networks [32]. NFI family members can influence other transcription factors’ DNA-binding ability while interacting with major chromatin remodelers. These properties suggest that NFIs may function as potential pioneer factors, thereby influencing a broad spectrum of cellular processes [32,33]. TBT-induced ovarian virilization in female chicken embryos resulted in *NFIB* upregulation. Differential expression of NFIB has been observed during early embryonic development in both mice and turtles, with distinct patterns observed between the sexes [34]. Both *NR2F1* and *NFIB* are transcription factors that play critical regulatory roles in early embryonic development [27,35]. Previous studies demonstrated their involvement in primary sex determination and gonadal differentiation [27,28,32]. *SP1*, *SP3* and *EGR1* have been validated as *DMRT1* transcription factors in rat; notably [16], *EGR1* was identified as a *DMRT1* transcription factor by the intersection of predictions from hTFtarget, JASPAR, ENCODE, ChEA and GTRD. These factors also obtain high predicted binding scores in the chicken *DMRT1* promoter region, consistent with *DMRT1* conservation [36]. We therefore propose that *SP1*, *SP3* and *EGR1* may likewise participate in the transcriptional regulation of chicken *DMRT1*. However, compared with *SP1*, *SP3* and *EGR1*, the predicted binding regions of *NR2F1* and *NFIB* on the chicken *DMRT1* promoter are distinct: *NR2F1* is located closer to the initiator element, and *NFIB* is nearer the CCAT-box. These findings suggest that *NR2F1* and *NFIB* may represent prime candidate transcriptional regulators of *DMRT1*.

Progress has been made on the key genes and molecular mechanisms related to sex determination and differentiation in poultry. The DMRT1 gene is the most upstream key gene involved in determining male sex, and it is specifically expressed in male embryos, with its deletion associated with sex reversal [11]. Single nucleus RNA-seq (snRNA-seq) of chicken embryonic gonads at E3.5, E4.5, and E5.5 revealed that cells marked by *DMRT1* and *NR5A1* were preferentially activated during the sex determination process. DMRT1 drives the fate of support cells during chicken testis development by altering chromatin accessibility [12]. Using CRISPR-Cas9–based monoallelic targeting, chromosomally male (ZZ) chickens with a single functional copy of *DMRT1* were generated, and they developed ovaries but not testes. This demonstrates that *DMRT1* dosage is a key switch in sex determination in birds and essential for testicular development [37]. Estrogen production is another key factor in primary sex determination in chickens, and it is also associated with *DMRT1* expression [37]. *DMRT1* expression is an early marker of testicular development in chicken embryos. In female gonads, over-expression of *DMRT1* can induce the male sex by upregulating *HEMGN*, *SOX9*, and *AMH* while downregulating the female gene underlying aromatase production [38]. In summary, *DMRT1* drives genital ridge differentiation in a dose-dependent manner, thereby determining gonadal sex differentiation in chickens.

Thus, the high expression of the transcriptional activators *NFIB* and *NR2F1* in the early gonads (E3.5) may lead to subsequent high expression of *DMRT1* (at E4) by binding to the *DMRT1* promoter region. This finding suggests that the early differential expression of NFIB and *NR2F1* between sexes could be the cause of the subsequent differential expression of *DMRT1*, which then triggers gonadal differentiation via dosage effects.

In summary, transcriptome sequencing of male and female chicken embryonic gonads at the E3.5 stage revealed several genes related to chicken sex determination during the early undifferentiated stages of sex development, including *SMAD2Z*, *FREM1*, *NR2F1*, *SEMA6A*, *NFIB*, *RNF165*, *SMAD7B*, *SMAD2W*, *SPIN1W*, and *HINTW*. These genes are potential candidates for determining chicken embryonic gonad differentiation. Among them, *NR2F1* and *NFIB* are transcriptional activators that present corresponding cis-acting elements in the *DMRT1* promoter region. These two transcription factors exhibited differential expression upstream of the male-specific DMRT1 gene, suggesting that they may act as switches to regulate the expression of the DMRT1 gene. Our study provides empirical support for the Z chromosome dosage hypothesis while identifying critical genes and potential regulatory mechanisms underlying avian sex determination in chickens. These findings establish a framework for advancing vertebrate evolutionary studies and elucidating the molecular basis of sex determination/differentiation. Additionally, they offer both conceptual foundations and practical applications for avian sex control technologies and livestock breeding improvement.

## 4. Materials and Methods

### 4.1. Sample Collection

This study utilized white Leghorn eggs provided by Experimental Chicken Farm of China Agricultural University (Beijing, China). All animal-related procedures were conducted in accordance with protocols approved by the Animal Welfare Committee of China Agricultural University (Permit Number: XK622). Eggs of uniform size and shape were selected and incubated under standard conditions (37.8 °C, 60% relative humidity, with automatic turning every 12 h. Embryonic gonadal ridges were collected at seven developmental stages (3.5, 4.0, 5.0, 6.0, 7.0, and 9.0 days post-incubation). At each time point, embryos were sexed by molecular genotyping, with 15 male and 15 female embryos collected per stage, yielding a total of 180 samples (6 stages × 30 embryos/stage). Embryonic gonadal crests were collected at 3.5, 4.0, 5.0, 6.0, 7.0, and 9.0 days of incubation. Only the 3.5-day samples were used for transcriptome sequencing. For RNA-seq, the 15 male and 15 female gonadal ridges at 3.5 days were pooled into three biological replicates per sex, with each replicate comprising tissue from five embryos (resulting in 3 male pools and 3 female pools; 6 pooled samples sequenced). The same pooling scheme (three biological replicates per sex, each pool containing tissues from five embryos) was used for RT-qPCR validation of selected DEGs at 3.5 days. For temporal *DMRT1* expression analysis by RT-qPCR, all collected time points (3.5, 4.0, 5.0, 6.0, 7.0 and 9.0 days) were analyzed using the same design: three biological replicates per sex at each time point, with each replicate pool comprising five embryos. Tissue samples were frozen at −80 °C until processing.

### 4.2. Sex Determination of Chicken Embryo

Genomic DNA was extracted from trunk tissue using a Tiangen DNA Extraction Kit (TIANGEN, Beijing, China, DP304-03). Simultaneously, DNA was extracted from the trunk tissue of chick embryos for sex identification using the primers 2550F/2718R, as described by Fridolfsson et al. [39]. Polymerase chain reaction (PCR) amplification of the CHD1 sequence on the chicken sex chromosome was performed. The primer sequences for amplifying the CHD1 gene regions are as follows: 2550F: 5′-GTTACTGATTCGTCTACGAGA-3′; 2718R: 5′-ATTGAAATGATCCAGTGCTTG-3′. The 15 μL PCR volume contained 7.5 μL of 2X M5 HiPer plus Taq HiFi PCR mix (with blue dye) V. 2 (Mei5 Biotechnology, Beijing, China), 5.9 μL of DNase-free water, 0.3 μL each of forward and reverse primers (10 μmol/L), and 1 μL of DNA (approximately 300 ng). PCR products were immediately electrophoresed on a 1% agarose gel at 120 V for 30 min. The target bands were visualized and sexed using a gel imaging system.

### 4.3. RNA Isolation and cDNA Preparation

After sex determination, five samples of the same sex were pooled to extract RNA from the ipsilateral embryonic gonadal tissue at each time of incubation, resulting in three pools for each sex. Total RNA was extracted from tissues using TRIzol reagent (Invitrogen, Carlsbad, CA, USA) and digested with DNase I (Takara Bio Inc., Otsu, Japan) to avoid genomic DNA contamination. The concentration and integrity of total RNA were determined via spectrophotometry (Nanodrop, NanoDrop Technologies, Wilmington, DE, USA) and gel electrophoresis, respectively. Total cDNA was synthesized using an ImProm II Reverse Transcriptase Kit (Promega, Madison, WI, USA).

### 4.4. RNA-Seq and Bioinformatics Analysis

Male and female RNA samples from incubation to 3.5 days were subjected to transcriptome sequencing to compare differences in gene and mRNA expression between the two sexes. RNA-seq analysis of the samples was performed by Frasergen Information Co., Ltd. (Wuhan, China). Raw RNA-seq data were deposited in the National Center for Biotechnology Information (NCBI)’s Sequence Read Archive (SRA) database under accession number PRJNA1141117. Clean reads were generated by removing adapters and low-quality reads from the raw data using SOAPnuke v2.1.0 (BGI, Shenzhen, China) [22] and aligned to the chicken genome using bowtie2 v 2.3.5 (Johns Hopkins University, Baltimore, MD, USA) [40]. Expression levels were normalized to fragments per kilobase of transcript per million mapped fragments (FPKMs) using RSEM v1.3.4 software. Differentially expressed genes (DEGs) were identified using DESeq2 v1.32.0 [41]. Enrichment analyses, including Gene Ontology (GO) and Kyoto Encyclopedia of Genes and Genomes (KEGG) (criteria: FDR < 0.05, (|log2FC| > 1)), were performed using the R package clusterProfiler (version 4.14.4) [42]. Enrichment analysis charts and GSEA plots were generated using the real-time interactive online data analysis platform OmicShare [43] (http://www.omicshare.com, accessed on 25 July 2024).

### 4.5. Quantitative Real-Time PCR (qRT-PCR)

The cDNA samples from incubation to 3.5 days were used for qRT-PCR validation of DEGs. The samples from incubation to 3.5, 4.0, 5.0, 6.0, 7.0, and 9.0 days were used to validate the relative expression levels of *DMRT1* at different stage points. qRT-PCR was performed using the SYBR fluorescent dye method. For each sample, 2 μg of total RNA was reverse-transcribed to generate cDNA using a FastKing RT Kit (TIANGEN, Beijing, China). The 20 μL qRT-PCR volume contained 10 μL of 2 × Universal SYBR Green Fast qPCR Mix (ABclonal, Wuhan, China), 8 μL of RNase-free water, 0.5 μL each of forward and reverse primers (10 μmol/L), and 1 μL of cDNA (approximately 300 ng). qRT-PCR was performed using a CFX96 Real-time System (Bio-Rad, Hercules, CA, USA). The expression levels of the coding genes were normalized to those of β-actin. Primer sequences were designed using the Primer-BLAST tool in the NCBI database and are listed in Table S1. Gene relative expression levels were analyzed and graphed using GraphPad Prism 9. All data are presented as the mean ± standard deviation (SD), and statistical analysis was performed using an unpaired two-tailed *t*-test. Statistical significance was defined as *p* < 0.05 (*).

### 4.6. Prediction of Binding Sites for Transcription Factors NFIB and NR2F2

The first 2100 bp of the chicken DMRT1 gene (NCBI Reference Sequence: NC_052572.1) represent the promoter region and were retrieved from the NCBI database. The Eukaryotic Promoter Database (EPD; https://epd.expasy.org/cgi-bin/epd/get_doc?db=ggEpdNew&format=genome&entry=DMRT1_1, accessed on 15 September 2025) [44] was used to predict regulatory elements in the chicken *DMRT1* promoter. We used the integrated tool TFTF [45] to predict transcription factors of human *DMRT1* by querying the hTFtarget, JASPAR, ENCODE, ChEA, and GTRD databases, and identified candidate key *DMRT1* transcription factors from the intersection of these results. The JASPAR database (http://jaspar.genereg.net/, accessed on 15 September 2025) was used to predict potential transcription factor-binding sites for *NFIB* and *NR2F2* within the core promoter region of the DMRT1 gene, with a relative score threshold set to 0.75. In addition, all transcription factors and their potential binding sites within the *DMRT1* core promoter with a relative score > 0.80 were identified using JASPAR.

## Figures and Tables

**Table 1:** Transcriptome data statistics.

Sample	Clean Reads	Mapped Reads (%)	Uniq Mapped Reads (%)	Expressed_Gene	Q30 (%)	GC (%)
Female1	21,999,587	91.61	83.7	18,302	92.4	47.3
Female2	20,677,927	91.34	81.7	18,160	91.3	48.4
Female3	22,500,482	93.19	84.26	18,334	92.5	46.4
Male1	20,478,251	90.95	82.29	18,564	91.5	47.2
Male2	22,323,360	91.21	78.58	18,274	91.1	49.0
Male3	21,026,280	91.47	82.69	18,210	91.7	48.0

## Data Availability

The data presented in this study are available in this article and Supplementary Materials.

## Supplementary Materials

The following supporting information can be downloaded at: https://www.mdpi.com/article/10.3390/ijms26199575/s1.
