# Supplementary material for: Identification of Key Differentially Expressed Genes During Early Sex Determination in Chicken Embryos
Source: Int J Mol Sci. 2025 Oct 1;26(19):9575. doi: 10.3390/ijms26199575 (PMC12524454; doi:10.3390/ijms26199575)
Supplement: Supplementary file 1 [file ijms-26-09575-s001.zip › Figure S1.pdf]

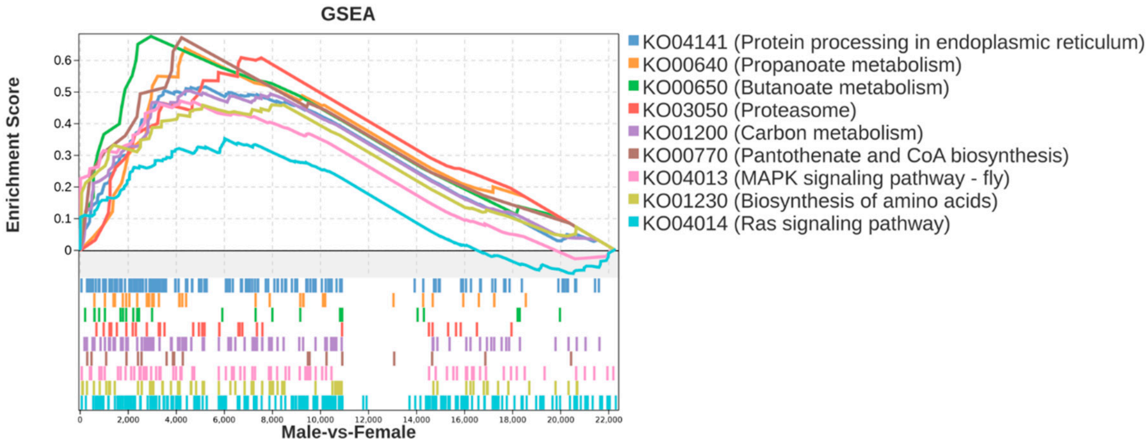

Figur S1. GSEA of selected KEGG pathways in DEGs between male (ZZ) and female (ZW) embryonic gonads at E3.5 (Gallus gallus).
